# Corpus callosum in children with neurodevelopmental delay: MRI standard qualitative assessment *versus* automatic quantitative analysis

**DOI:** 10.1186/s41747-023-00375-4

**Published:** 2023-10-13

**Authors:** Natacha Mandine, Elsa Tavernier, Till Hülnhagen, Bénédicte Maréchal, Tobias Kober, Clovis Tauber, Marine Guichard, Pierre Castelnau, Baptiste Morel

**Affiliations:** 1 Pediatric Radiology Department, CHRU of Tours, Clocheville Hospital, Tours, France; 2 Clinical Investigation Center, INSERM 1415, CHRU Tours, Tours, France; 3 Advanced Clinical Imaging Technology, Siemens Healthineers International, Lausanne, Switzerland; 4 Department of Radiology, Lausanne University Hospital and University of Lausanne, Lausanne, Switzerland; 5 LTS5, École Polytechnique Fédérale de Lausanne (EPFL), Lausanne, Switzerland; 6 UMR 1253, iBrain, Université de Tours, Inserm, Tours, France; 7 Pediatric Neurology Department, CHRU of Tours, Clocheville Hospital, Tours, France

**Keywords:** Brain, Child, Corpus callosum, Segmentation, Magnetic resonance imaging

## Abstract

**Background:**

The corpus callosum (CC) is a key brain structure. In children with neurodevelopmental delay, we compared standard qualitative radiological assessments with an automatic quantitative tool.

**Methods:**

We prospectively enrolled 73 children (46 males, 63.0%) with neurodevelopmental delay at single university hospital between September 2020 and September 2022. All of them underwent 1.5-T brain magnetic resonance imaging (MRI) including a magnetization-prepared 2 rapid acquisition gradient echoes − MP2RAGE sequence. Two radiologists blindly reviewed the images to classify qualitatively the CC into normal, hypoplasic, hyperplasic, and/or dysgenetic classes. An automatic tool (QuantiFIRE) was used to provide brain volumetry and T1 relaxometry automatically as well as deviations of those parameters compared with a healthy age-matched cohort. The MRI reference standard for CC volumetry was based on the Garel et al. study. Cohen κ statistics was used for interrater agreement. The radiologists and QuantiFIRE’s diagnostic accuracy were compared with the reference standard using the Delong test.

**Results:**

The CC was normal in 42 cases (57.5%), hypoplastic in 20 cases (27.4%), and hypertrophic in 11 cases (15.1%). T1 relaxometry values were abnormal in 26 children (35.6%); either abnormally high (18 cases, 24.6%) or low (8 cases, 11.0%). The interrater Cohen κ coefficient was 0.91. The diagnostic accuracy of the QuantiFIRE prototype was higher than that of the radiologists for hypoplastic and normal CC (*p* = 0.003 for both subgroups, Delong test).

**Conclusions:**

An automated volumetric and relaxometric assessment can assist the evaluation of brain structure such as the CC, particularly in the case of subtle abnormalities.

**Relevance statement:**

Automated brain MRI segmentation combined with statistical comparison to normal volume and T1 relaxometry values can be a useful diagnostic support tool for radiologists.

**Key points:**

• Corpus callosum abnormality detection is challenging but clinically relevant.

• Automated quantitative volumetric analysis had a higher diagnostic accuracy than that of visual appreciation of radiologists.

• Quantitative T1 relaxometric analysis might help characterizing corpus callosum better.

**Graphical Abstract:**

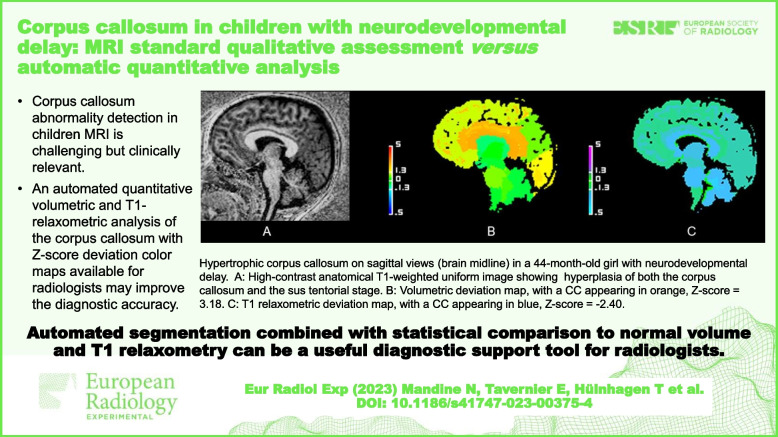

## Background

The corpus callosum (CC) is a key structure of the midline brain structure. It provides interhemispheric communication, including distant regions of the cerebral cortex [1]; it also participates in several cognitive functions [2] and in the integration of sensory-motor information. The normal anatomy of the CC from anterior to posterior is divided into the rostrum, the genu, the body, the isthmus and the splenium [3, 4], its normal embryological development takes place during the 12th to the 20th week of gestation [5].

The morphology of the CC changes structurally during childhood and all lifelong [6]: its thickness and signal intensity have been reported to reflect the density and the myelination of the white matter fibers, showing a positive correlation with intelligence [7]. Due to its central location and role, congenital or acquired anomaly of the CC can have drastic effects on brain development and its function. However, only the definition of complete agenesis of the CC is consensual, confirmed with indirect signs observed on ultrasound or MRI [8]. The partial agenesis of the CC, due to a lack of visualization of one of its parts, is more difficult to diagnose despite well-defined criteria [9]. All other morphological abnormalities of the CC, *i.e.,* reduced (hypoplasia) or increased thickness (hyperplasia), or an abnormal orientation of the whole structure or of one of its parts, have variable definitions in the fetal CC pathology literature [10]. The presence of CC anomalies in the prenatal period is often associated with a poor prognosis, especially if other cerebral anomalies are present [9, 11, 12]. In the postnatal period, a reduced or increased CC thickness is associated with a wide spectrum of clinical abnormalities [13] such as developmental disorders, epilepsy, or neurocutaneous syndromes such as neurofibromatosis type 1 [14].

The normal size and thickness of the prenatal CC has been reported using two- [15] and three-dimensional ultrasound [16, 17] as well as two-dimensional MRI [18]; these data are also available for the whole childhood phase [15, 19, 20]. In MRI, the volumetric analysis of the CC by the radiologist may require the use of measurements in case of doubt about its normality. However, the measurement methods vary widely with different normality thresholds and require an experienced operator to be reproducible. The determination of the reference standard in MRI was based on the study performed by Garel et al. [19], who established a biometric classification of CCs, the reproducibility of which was limited in current practice due to a large number of measurements.

To address this problem, an automated MRI-based segmentation of the midline brain structures relying on the magnetization prepared 2 rapid acquisition gradient echoes (MP2RAGE) sequence was previously developed [21] to provide CC volumetry and T1 mapping, the latter also giving an indication of the microstructural tissue status. In addition, the method incorporates a comparison with normative values of both volumes and T1 values including a spatial visualization of deviations from the norm using *Z*-score maps.

The study aimed to compare the diagnostic accuracy of radiological standard reading with an automatic quantitative CC assessment of both volumetry and T1 relaxometry in children with neurodevelopmental delay.

## Methods

### Study population

Following the Standards for Reporting of Diagnostic Accuracy Studies − STARD 2015 recommendations [22], we have prospectively and consecutively studied 75 consecutive children with neurodevelopmental delay aged 1 to 16 years who underwent a 1.5-T brain MRI examination at the Tours university hospital between September 2020 and September 2022. Patients with complete CC agenesis (*n* = 0) were initially excluded. In addition, two children were excluded due to severe motion artefacts. Thus, the included population consisted of 73 children (46 males, 63.0%). The mean age was 50 ± 37 months, mean standard deviation (range 12–167). Approval by the local Ethics Committee in Human research (n°2022_135) was obtained prior to starting the study.

### MRI protocol

All patients were scanned at 1.5-T scanner (MAGNETOM Sola, Siemens Healtheeners, Erlangen, Germany) using a standard 20-channel head coil. Intrarectal pentobarbital (5 mg/kg) was administrated to young children requiring sedation or general anesthesia when required. Whole-brain T1-weighted imaging and simultaneous T1 mapping were achieved with the MP2RAGE sequence using acquisition parameters tailored to pediatric applications (spatial resolution 1.33 × 1.33 × 1.33 mm^3^; field of view 256 × 192 mm^2^, inversion time_1_ /inversion time_2_ 643/1,960 ms; flip angles 5°, 6°, repetition time 5,000 ms, echo time 2.83 ms, acquisition time 5 min).

### Reports and deviation maps creation

An automated brain segmentation was performed using a dedicated MP2RAGE processing pipeline based on the MorphoBox research application [23] with a previously reported adaptation of its templates [21]. A volumic segmentation of a total of 38 anatomical brain structures was obtained according to the standard anatomical nomenclature, including the CC [24]. This adapted MorphoBox pipeline together with the regional T1 mapping analysis reported in [21] was integrated into a research application referred to in this work as “QuantiFIRE”. This integration enabled the fully automatic processing of the input, producing a volumetric and regional T1 mapping report embedded in a DICOM file which was automatically transferred to the PACS. The segmentation quality index appeared on the first page of the analysis report and enabled ensuring the quality of the segmentation with a range from 0 to 1. A value < 0.7 was considered insufficient.

Automated brain volumetric and T1 relaxometry results were compared with normative ranges and displayed on a deviation color map and in a tabulated Digital Imaging and Communication in Medicine − DICOM report, both available on our Picture Archiving and Communication System. CC *Z*-score values above 1.3 or below -1.3 were considered pathological, corresponding to the 10th and the 90th percentile, as established in a prior study [21].

### CC analysis: radiologists’ interpretation, automated analysis and reference standard

A radiology resident and a senior pediatric radiologist with 10 years of experience blindly reviewed the MRI exams in order to classify the CC visually into normal (normal shape and thickness), hypoplasic (normal shape but thinner), or hyperplasic (normal shape but thicker) and/or dysgenetic (irregular shape) classes on the midsagittal plane on the three-dimensional MP2RAGE sequence.

**Fig. 1:**
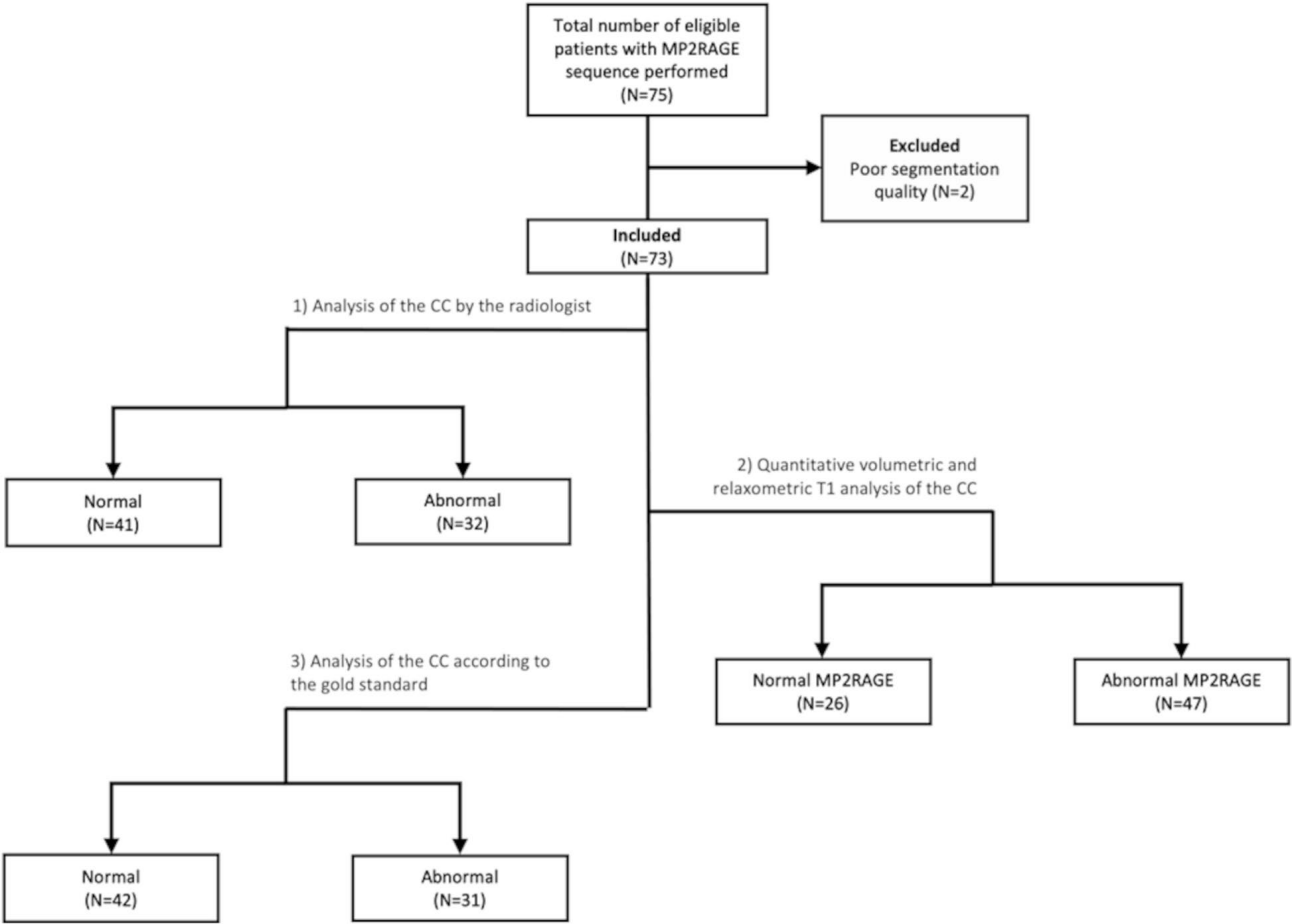
Patient flow chart and analysis steps

Based on the volumetric and T1 mapping *Z*-scores of the CC, cases were classified with respect to volumetry (normal, hypotrophic, or hypertrophic) and T1 deviations (normal, decreased, and increased T1 values). The reference standard determination of the CC size was obtained using the Garel et al. biometry classification [19]. The patient’s flow chart and the analysis steps are reported in Fig. 1.

The classification of each CC is listed in Table 1.

**Table 1:** Classification of the corpus callosum in the different groups

Groups		Results
Reference standard	Normal volume	42
	Increased volume	11
	Decreased volume	20
Radiologist	Normal volume	41
	Increased volume	13
	Decreased volume	12
	Dysgenetic CC	7
QuantiFIRE volumetric measures	Normal volume	40
	Increased volume	13
	Decreased volume	20
QuantiFIRE relaxometric measures	Normal T1	47
	Increased T1	18
	Decreased T1	8

### Statistical analysis

We calculated the interrater Cohen κ coefficient between the two radiologists. The radiologists and the QuantiFIRE research application’s diagnostic accuracy for each CC abnormality were calculated and compared with the reference standard method. All estimates were given with their 95% confidence interval. We compared the areas under paired receiver operating characteristic curves with the Delong test [25].

## Results

### Study population

**Fig. 2:**
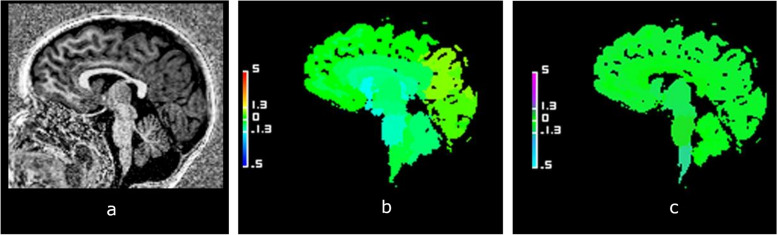
Example of a case of normal corpus callosum (CC). Sagittal view of the brain midline in a 3-year-old girl with neurodevelopmental delay. **a** High-contrast anatomical T1-weighted uniform image showing a normal CC. **b** Normal volumetric deviation map, with a CC appearing in green, Z-score = 0. **c** Normal T1 relaxometric deviation map, with CC appearing in green, Z-score = -0.52

**Fig. 3:**
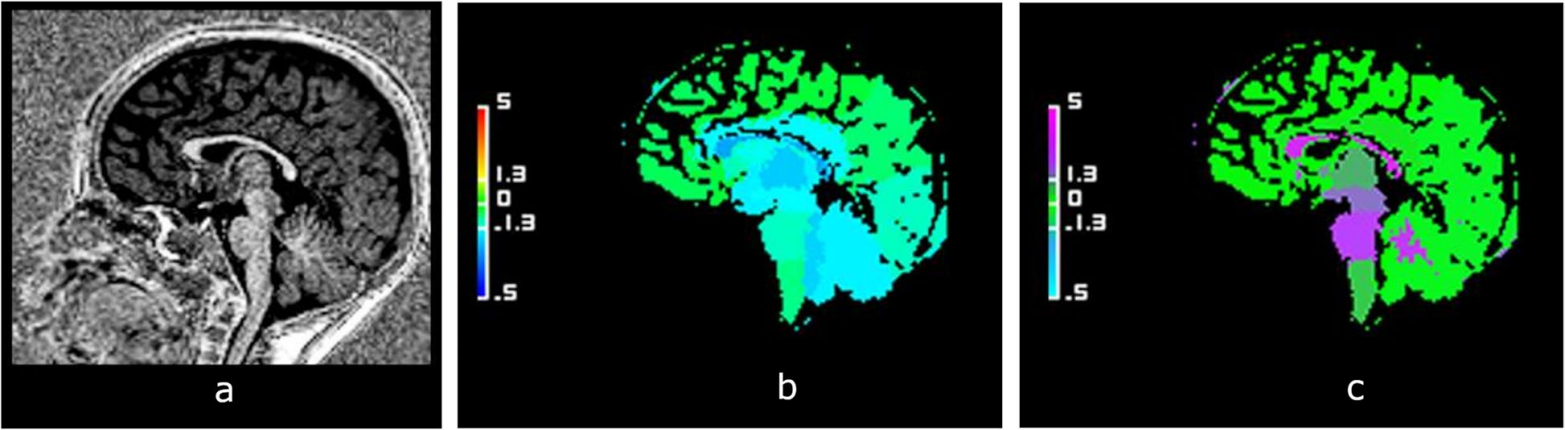
Example of hypotrophic corpus callosum (CC). Sagittal views of the brain midline in a boy aged 2 years and 11 months with neurodevelopmental delay. **a** High-contrast anatomical T1-weighted uniform image showing a hypoplasia of the corpus callosum. **b** Abnormal volumetric deviation map, with a CC appearing in light blue, *Z*-score = -2.86. **c** Increased T1 relaxometric deviation map, with a CC appearing in purple, *Z*-score = 3.20

**Fig. 4:**
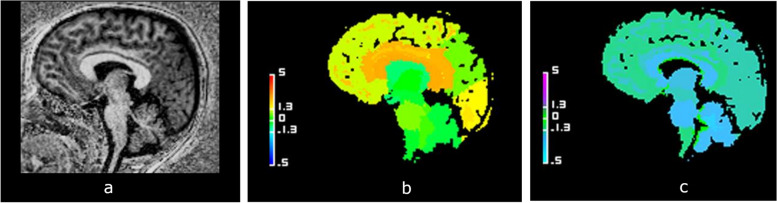
Example of hypertrophic corpus callosum (CC). Sagittal views of the brain midline in a 44-month-old girl with neurodevelopmental delay. **a** High-contrast anatomical T1-weighted uniform image showing a hyperplasia of both the corpus callosum and the sus tentorial stage. **b** Abnormal volumetric deviation map, with a CC appearing in orange, *Z*-score = 3.18. **c** Decreased T1 relaxometric deviation map, with a CC appearing in blue, *Z*-score = -2.40

**Fig. 5:**
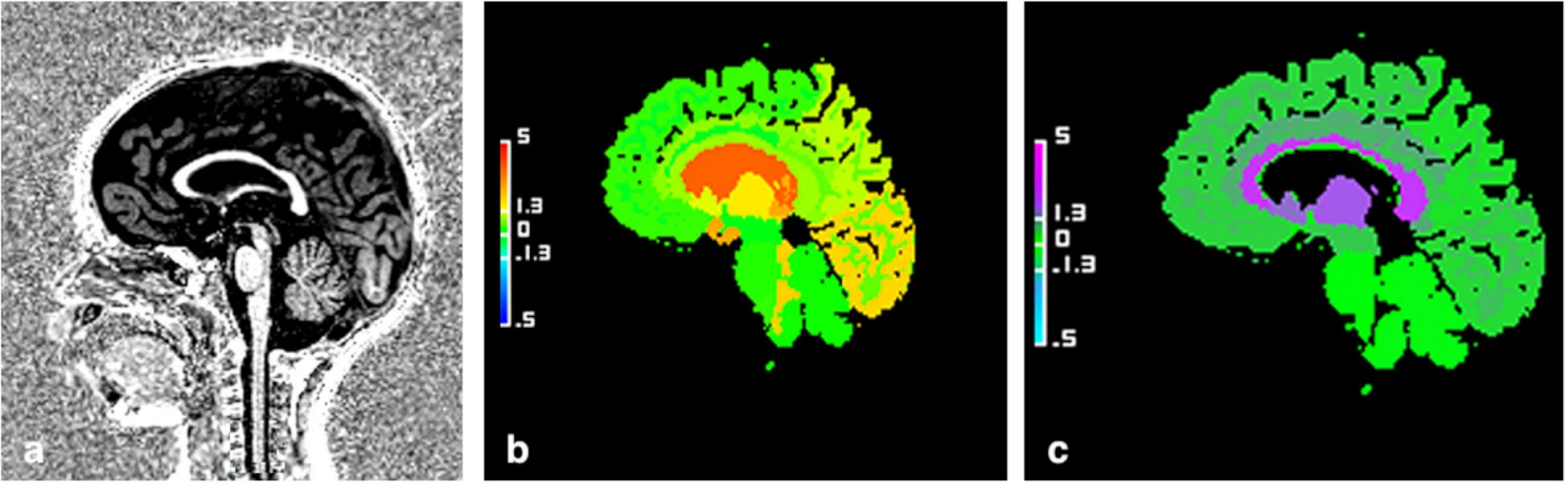
Example of a 2-year-old and 7-month-old child with neurodevelopmental disorders with an isolated T1 relaxometry anomaly. Midline sagittal reconstructions. **a** High-contrast uniform T1-weighted anatomical sequence showing a corpus callosum of normal shape and thickness. **b** Map of normal volumetric deviations, with CC appearing in green, *Z*-score = 0.49. **c** Map of abnormally increased T1 relaxometric deviations, with CC appearing purple, *Z*-score = 2.20

The volume of the CC was normal in 42 cases (57.5%), hypoplasic in 20 cases (27.4%), hyperplasic in 11 cases (15.1%), illustrated in Figs. 2, 3 and 4, respectively, according to the reference standard method. T1 relaxometry values of the CC were abnormal in 26 children (35.6%); either increased (18 cases, 24.6%) or decreased (8 cases, 11.0%). The number of children with normal brain volumetry and isolated abnormal T1 relaxometry values was 14 patients (Fig. 5), mainly males (*n* = 13; 92.3%). Focusing on the 7 dysgenetic CC cases diagnosed by the radiologists, one had both normal volume and T1 relaxometry values, four showed too low volumes (including three having too high T1 values) and two had normal volumes with too high T1 values.

### Diagnostic accuracy

The radiologists interrater Cohen κ coefficient was 0.91. The diagnostic accuracy of the radiologists and QuantiFIRE for classification of the CC volume is reported in Table 2. The diagnostic accuracy for QuantiFIRE was higher than that of the radiologists in patients with hypoplasic and normal CC. Delong’s test *p* values for the comparison of hypoplasia, normal and hypertrophic CC were 0.003, 0.003, and 0.274, respectively.

**Table 2:** The diagnostic accuracy of the radiologists and QuantiFIRE for classification of the CC volume

	Hypoplasia (*n* = 20)		Normal (*n* = 42)		hypertrophic CC (*n* = 11)	
	Radiologists	QuantiFIRE	Radiologists	QuantiFIRE	Radiologists	QuantiFIRE
Sensitivity	60 (36 to 81)	95 (75 to 100)	83 (69 to 93)	93 (81 to 99)	91 (59 to 100)	100 (72 to 100)
Specificity	92 (82 to 98)	98 (90 to 100)	71 (52 to 86)	97 (83 to 100)	95 (87 to 99)	97 (89 to 100)
Positive predictive value	75 (48 to 93)	95 (75 to 100)	80 (65 to 90)	98 (87 to 100)	77 (46 to 95)	85 (55 to 98)
Negative predictive value	86 (74 to 94)	98 (90 to 100)	76 (56 to 90)	91 (76 to 98)	98 (91 to 100)	100 (94 to 100)
False positive rate	25 (7 to 52)	5 (0 to 25)	20 (10 to 35)	2 (0 to 13)	23 (5 to 54)	15 (2 to 45)
False negative rate	14 (6 to 26)	2 (0 to 10)	24 (10 to 44)	9 (2 to 24)	2 (0 to 9)	0 (0 to 6)
Incidence	27 (18 to 39)		58 (45 to 69)		15 (8 to 25)	

Data are given as percentages with their 95% confidence intervals in parentheses

## Discussion

Corpus callosum abnormalities were frequent in our cohort of children with neurodevelopmental delay, manifesting either in volumetry and/or in T1 relaxometry deviations.

A non-negligible number of hypoplasic CC was missed by radiologists (8/20, 40%), despite a high interrater reliability, because only visual inspection was used. With a more appropriate and accurate approach they would not have been missed, but with longer time for images analysis with respect to automatic software. Indeed, the interpretation of CC shape and thickness by the radiologists is time demanding. The wrong appreciation of the size of the pediatric brain due to the partial subjectivity of the visual analysis by radiologists was shown by Serru et al. [26]. This is similar to the difficulty in distinguishing brain signal intensity variation by the human eye [27], the heterogeneity in the volume analysis constitutes an invitation to use automated software analysis to help radiologists not to be mistaken. In our study, the radiologists were mainly able to detect the most severe thickness variations, which were confirmed by the abnormal *Z*-scores provided by the QuantiFIRE software. Focusing on the volumetric analysis, the software had a higher diagnostic accuracy compared with that of the radiologists for normal and hypoplastic CC. It could help radiologists to obtain in a few seconds an extensive volumetry analysis, with high reproducibility at 3 T [28]. As the segmentation relied on a pediatric tailored atlas-based Morphobox pipeline, based on a normal anatomical brain, the QuantiFIRE software might have been led to default in some pathological cases or severe variation of the shape of the CC. The same MP2RAGE sequence was used at 1.5 T to obtain our pediatric normative data.

However, to diagnose any CC hypo or hyperplasia efficiently is a priority, and to look for any other associated brain anomaly that might help identify an underlying pathology [13]. Guo et al. [29] observed that the total brain volume in patients with dysgenesis of the CC was normal and that left hemisphere gyrification index abnormality was a specific predictive factor of CC dysgenesis. Alteration of the CC morphology has been reported in children under 2 years with autism [30], during youth in children with psychosis spectrum [31], in attention deficit hyperactivity disorder [32] or in dyslexia [33]. Difficulty in diagnosing hypoplastic or hypertrophic CC might be partially explained by the variation of the speed of growth during childhood [34], associated with selective increase in thickness of some part of the CC [35] and individual variability [36]. Depending on the time of the MRI acquisition and of the age of the children, the anomaly of development of the CC requires a radiologist’s expertise to be identified.

Concerning the definition of normal biometric findings based on two- or three-dimensional [16] ultrasound or MRI in both fetuses and in children, a wide heterogeneity of methods exists, varying in the number and the orientation of the measures [6, 15, 18–20, 31, 37]. A dedicated automated algorithm enables multiple measurements of the CC to obtained but with the potential inconvenience of complicating the availability and the use of the method when a lot of measures have to be compared with reference ranges [32]. It highlights the interest of developing automatic quantitative evaluation. The review of Rosenbloom et al. [38] also observed the need for the homogenization and clear definition of the criteria in order to determine normal biometric ranges [38]. Many studies have defined different thresholds to consider a pathological size of the CC: from the 3rd [18, 19], 5th [15, 17] to the 10th centile [9, 39], while stricter thresholds increase specificity.

The additional quantitative characterization provided by the automated assessment with the QuantiFIRE software is the T1 relaxometry, which includes a comparison with normative values. Both this and the morphometry results were directly available in the radiological reading environment. We interpreted the findings so that a normal CC T1 relaxometry was complementary evidence of the normality of the CC. Kühne et al. [40] have noticed also that the CC has the fastest myelination rate in the brain and was a reflection of the normal brain maturation. The measurement of T1 relaxometry values from different brain structures is an emerging technique. Published results suggest that the T1 relaxometry value reflects the normality of myelination in the brain [40] and cerebral tissues in general [41]. Although this requires future confirmation, particularly with anatomopathological correlations, it is possible that abnormal relaxometry values may be associated with organic abnormalities of cerebral tissue, potentially affecting brain function. This discovery is particularly interesting in the context of studying neurodevelopmental disorders. Conversely, we found that approximately one third of the children showed abnormal T1 values. This quantitative analysis could help to understand and to identify subtle non-morphologic abnormalities of the CC tissue and potentially better characterize neurodevelopmental delay better. In our study, the whole CC was segmented and its median T1 value was available. To be more precise and efficient, a segmentation of the CC into anatomical sub-compartments could be a way to depict some regional variation better, as described by Hofer et al. [42]. Brain T1 relaxometry still requires some research to determine its significance clearly. Eminian et al. [41] have considered that T1 relaxometry and apparent diffusion coefficient were complementary quantitative values that both enable the study of the brain maturation and growth with two different aspects. In our cohort, we observed that a quarter of the patients had increased T1 relaxometry values. A potential cause could be a myelination retardation or another brain tissue pathology, particularly if these children were under 2 years old.

The CC is a major structure of the midline brain, and its volume variation might explain several neurological symptoms. We have to see that a prenatal hypoplasia or a complete or partial agenesis of the CC diagnosis was associated with severe neurological outcomes from developmental delay and epilepsy [43]. Further to that, it is also very important to diagnose hyperplasia of the CC, either prenatally [39, 44] or postnatally [13]. This is because a genetic or syndromic association has been reported, such as megalencephaly-capillary malformation-polymicrogyria syndrome [45], in which in half of the cases a thick CC was described, or in neurofibromatosis [14, 29]. A particular improvement should be made in this context to take care of children better, with the help of an automatic comparison of CC size.

A limitation of our study is the limited number of cases and the need for further correlation with genetic or syndromic pathologies. It is important to acknowledge that the reference standard was not always employed in clinical practice. Therefore, we chose to conclude our study by focusing on the radiologists’ initial analysis, as it is more applicable to routine practice and can be extrapolated more easily.

It would be interesting in a next study to compare the volumetric results of the CC of each patient, to the total cerebral volume, and to consider potential cranio-facial malformations. The segmentation of the CC was global with our automated tool. We do not distinguish the different segments and the variations in volume and myelination. However, the children included in our study were mostly over 2 years old. We can consider that myelination was normally complete. Our normative values of relaxometry evolve according to age between 1 and 15 years. It would indeed be interesting to segment each subpart of the CC more precisely and to obtain normal values. This could be done in the future*.* To confirm that the CC volume analysis is valid, a visual validation of the segmentation should be performed before considering the quantitative results and their interpretation. Further research on the brain volumetry and T1 relaxometry is still mandatory to understand the physiology and pathological process better. Indeed, the value of T1-relaxation measurements is difficult to interpret without correlations with clinical data.

We found that a number of boys of our cohort showed isolated abnormal T1 values. As the normal reference values used for volumetry and T1 relaxometry were based on a limited population of children, we must confirm the results by a larger prospective multicentric study to validate the possibility of extending the use of the established norms to different centers. With a larger cohort, we will have to verify in a future study whether our normative values should consider gender as a covariate, as some transient discrepancy in size between boys and girls has been previously reported [3, 15, 46]. These were however not observed in T1 relaxometry [47]. After this verification, it should be investigated whether there may be a specific underlying pathology responsible for the abnormal T1 relaxometric values.

In conclusion, an automated quantitative analysis of the midline brain structures such as the CC with *Z*-score deviation color maps available for radiologists may improve the diagnostic accuracy of subtle abnormalities.

## Data Availability

Not applicable.

## Abbreviations

CC: Corpus callosum
MP2RAGE: Magnetization prepared 2 rapid acquisition gradient echoes
MRI: Magnetic resonance imaging
